# The genome and proteome of a virulent *Escherichia coli *O157:H7 bacteriophage closely resembling *Salmonella *phage Felix O1

**DOI:** 10.1186/1743-422X-6-41

**Published:** 2009-04-20

**Authors:** Andre Villegas, Yi-Min She, Andrew M Kropinski, Erika J Lingohr, Amanda Mazzocco, Shivani Ojha, Thomas E Waddell, Hans-Wolfgang Ackermann, Dianne M Moyles, Rafiq Ahmed, Roger P Johnson

**Affiliations:** 1 Public Health Agency of Canada, Laboratory for Foodborne Zoonoses, 110 Stone Road West, Guelph, ON, N1G 3W4, Canada; 2 Centre for Biologics Research, Biologics and Genetic Therapies Directorate, Health Canada, 251 Sir Frederick Banting Driveway, Tunney's Pasture, Ottawa, ON, K1A 0K9, Canada; 3 Department of Molecular & Cellular Biology, University of Guelph, Guelph, ON, N1G 2W1, Canada; 4 Pro-Lab Developments Inc, 200 Gerrard Street E, Suite 300, Toronto, ON, M5A 2E6, Canada; 5 Département de Biologie médicale, Faculté de médecine, Université Laval, Québec, QC, G1K 7P4, Canada; 6 Public Health Agency of Canada, National Microbiology Laboratory, Canadian Science Centre for Human and Animal Health, 1015 Arlington Street, Winnipeg, MB, R3E 3R2, Canada

## Abstract

Based upon whole genome and proteome analysis, *Escherichia coli *O157:H7-specific bacteriophage (phage) wV8 belongs to the new myoviral genus, "the Felix O1-like viruses" along with *Salmonella *phage Felix O1 and *Erwinia amylovora *phage φEa21-4. The genome characteristics of phage wV8 (size 88.49 kb, mol%G+C 38.9, 138 ORFs, 23 tRNAs) are very similar to those of phage Felix O1 (86.16 kb, 39.0 mol%G+C, 131 ORFs and 22 tRNAs) and, indeed most of the proteins have their closest homologs within Felix O1. Approximately one-half of the *Escherichia coli *O157:H7 mutants resistant to phage wV8 still serotype as O157:H7 indicating that this phage may recognize, like coliphage T4, two different surface receptors: lipopolysaccharide and, perhaps, an outer membrane protein.

## Findings

Bacteriophages (phages) are promising potential alternatives to antibiotics as therapeutics to reduce carriage of pathogens by food animals, thus preventing the spread of organisms such as *Escherichia coli *O157:H7 along the food chain. Our research has shown that a cocktail of virulent phages can eliminate *E. coli *O157:H7 from experimentally infected calves [1,2]. Phage V8, isolated originally from sewage [3] was renamed wV8 in our laboratory to indicate that it was obtained from the National Microbiology Laboratory (Winnipeg), and was included in the phage cocktail due to its complementary host range on common phage types (PTs) of *E. coli *O157:H7. Here we report on the genome and proteome of phage wV8, noting its very close similarity to the *Salmonella *phage Felix O1 [4-7].

**Figure 1:**
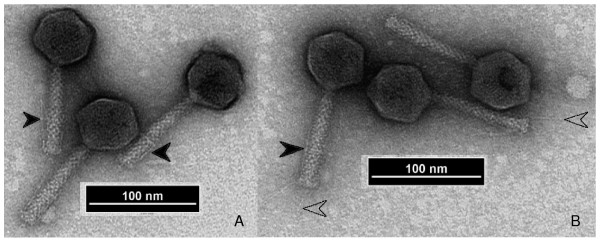
**Electron micrographs of phage wV8 showing typical myovirus morphology**. Open arrowheads (Figure 1B) point to extended tail fibres while filled arrowheads (Figures 1A, 1B) point to the more commonly observed folded tail fibres. A collar and neck can be seen on several of the particles.

Phage wV8, purified as described below, was negatively stained with 1% (w/v) uranyl acetate for 20 s and the particles were observed using a LEO912AB and a Philips EM 300 transmission electron microscope operating at 100 kV and 60 kV, respectively. Phage wV8 is a member of the *Myoviridae *and is morphologically identical to Felix O1 and related phages. Viral particles were morphologically intact and generally have extended tails (Figure 1). Measurement of 20 particles indicated phage wV8 has a head 70.4 nm in diameter and a tail 112.8 × 16.8 nm long. These closely resemble those reported for phage Felix O1, in which the head measured 73 nm in diameter and the noncontracted tail was 113 × 17 nm long [8]. Phage wV8 has a neck of 7 × 7 nm, a collar disk of 10 × 2 nm, and four fibres of 40 × 2 nm that are generally folded along the tail, but may become unfolded in some particles. Tails have transverse striations of 3 nm periodicity, but sometimes present a pattern of overlapping subunits.

For host range studies, phage wV8 was tested for lytic activity on reference strains of 12 common *E. coli *O157:H7 PTs, the entire ECOR collection [9] and 12 *Salmonella enterica *serovars. Lytic activity on the reference *E. coli *O157:H7 PT strains and the *Salmonella *serovars was determined at multiplicities of infection (MOI) of between 0.001 and 10 in broth cultures in microplates incubated for 5 h at 37°C before inspection for complete lysis (no visible turbidity). Bacteria showing no visible lysis at any MOI were considered resistant to phage wV8, while those showing complete lysis at MOIs of 10 or less were considered sensitive to phage wV8. Strains of the ECOR collection were tested as freshly seeded bacterial lawns on agar plates spotted with 20 μl of diluted phage wV8 containing 10^4^-10^6 ^pfu. After incubation for 18 h at 37°C, strains showing >50% lysis were considered sensitive. Phage wV8 is highly specific for *E. coli *O157:H7 strains, completely lyses the 12 most common *E. coli *O157:H7 (PTs) isolated in Canada [10] and has no lytic activity against any of the *Salmonella *strains (Table 1).

**Table 1:** Sensitivity and resistance of bacterial cultures to bacteriophage wV8

	**Bacteria**	**Sensitive to bacteriophage wV8**	**Resistant to bacteriophage wV8**
*E. coli *O157:H7	Reference strains of 12 common *E. coli *O157:H7 phage types^1 ^(1 strain/phage type)	All tested reference strains: phage types 1, 2, 4, 8, 14, 21, 23, 24, 31, 32, 33, 87.	None
Other *E. coli*	The ECOR collection^2^	ECOR 6 (O173:H-); ECOR 28 (O104:H2)	ECOR Strain No. 2, 3, 5, 7, 8, 11, 14–19, 21–27, 29–44, 46, 48–72
*Salmonella enterica*	2 serovars:	None	*S*. Anatum, *S*. Hadar, *S*. Heidelberg, *S*. Infantis, *S*. Kentucky, *S*. Meleagridis, *S*. Muenchen, *S*. Munster, *S*. Newport, *S*. Thompson, *S*. Typhimurium, and *S*. Schwarzengrund

^1 ^The 12 most common *E. coli *O157:H7 phage types that represent >93% of *E. coli *O157:H7 isolates phage typed in Canada in 1998–99 [10].

^2 ^The ECOR collection is a reference collection of 72 strains of *E. coli *that represents the genotypic diversity of *E. coli*, as determined by multilocus enzyme electrophoresis [9].

Phage wV8 was propagated on *E. coli *strain EC990779 (ECOR strain 6, O173:H), precipitated from clarified lysates using polyethylene glycol 8000 and purified through two rounds of CsCl equilibrium gradient centrifugation [11]. The DNA was isolated as described by these authors and subjected to pyrosequencing at the National Microbiology Laboratory (Winnipeg, MB). Prior to annotation, the genome was opened immediately upstream of the *rIIA *gene so that it could be directly compared with the sequence of Felix O1. The genome was annotated using Kodon (Applied Maths, Austin, TX) and a variety of online tools  including tRNAScan-SE [12] and ARAGORN [13] at their default setting. The GenBank accession number for this sequence is EU877232.

**Figure 2:**
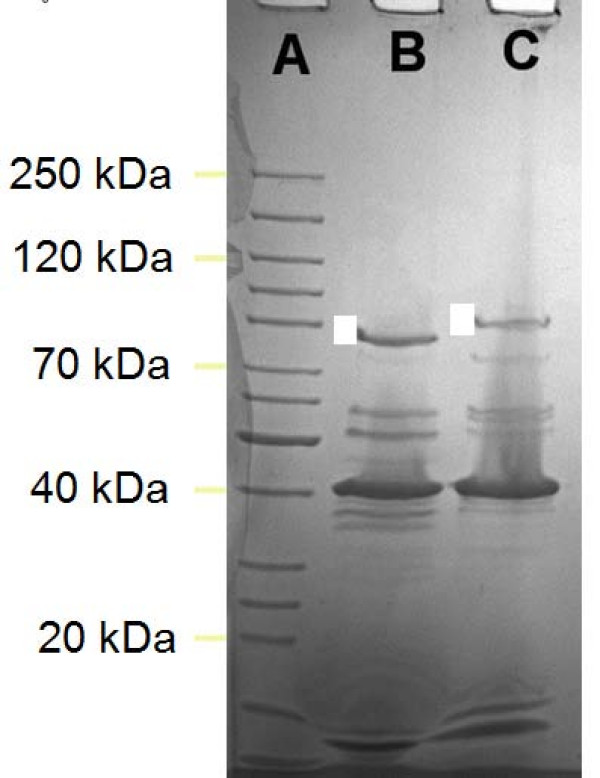
**Structural proteins of phages Felix O1 (lane B) and wV8 (lane C) revealed by SDS-PAGE**. Clear boxes are to the left side of the phage tail fibre protein bands.

The genome characteristics of wV8 (size: 88.49 kb, 38.9 mol%G+C, 138 ORFs, 23 tRNAs) closely resemble those of *Salmonella *phage Felix O1 (86.16 kb, 39.0 mol%G+C, 131 ORFs and 22 tRNAs) and, indeed many of the proteins have their closest homologs with those of Felix O1 (NC_005282). This is also substantiated by SDS-PAGE analysis of the structural proteins of wV8 and Felix O1 which show considerable similarity (Figure 2). The one notable exception lies in the largest proteins (wV8 Gp83, 91.5 kDa; and, Felix O1 orf184, 84.1 kDa), which bioinformatic analyses revealed to be the tail fibre proteins. Matrix-assisted laser desorption ionization quadrupole time-of-flight (MALDI QqTOF) MS analysis of the wV8 protein indicates that these two proteins are homologous.

Tandem mass spectrometric (MS/MS) measurements were performed to sequence all the trypsin-digested peptides in order to obtain the high confidence protein identification in the databases. Initial MS/MS search using Mascot  against NCBI databases retrieved a putative tail fibre protein from phage Felix O1 (NCBI: GI:38707850, NP_944923), where five sequences out of the observed 28 peptides were matched (m/z 775.427, 1361.735, 1764.869, 2019.015, 2215.053). Using a custom wV8-specific protein database, a thorough examination of all peptide sequences confirmed the protein assignment, with these five peptides providing 44.3% sequence coverage. Sequence alignment of the Felix O1 and wV8 tail fibre orthologs using ALIGN  revealed 65.7% identity.

Tail fibre proteins from related phages typically show strong sequence similarity at the N-termini, where the protein associates with the phage tail plate. The carboxy termini, associated with receptor interaction, vary considerably. With Felix O1 and wV8, we see a completely different type of relationship: four regions of similarity separated by regions of dissimilarity, with both the C- and N-termini conserved (see Additional file 1) [14].

Since Felix O1 is LPS-specific [15], we analyzed wV8-resistant mutants of *E. coli *O157:H7. An overnight broth culture of an *E. coli *O157:H7 strain was mixed with excess wV8 and incubated on plates for 24 h. Nine independent mutants were isolated and serotyped by the *E. coli *(VTEC) Reference Laboratory at the Laboratory for Foodborne Zoonoses. Approximately one-half of these still serotyped as O157:H7, while half were untypable (rough) indicating that this phage may recognize, like coliphage T4, two different surface receptors: lipopolysaccharide and, perhaps, an outer membrane protein.

**Figure 3:**
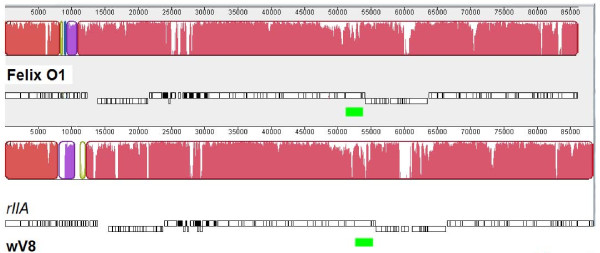
**Alignment, based upon BLASTN of the genome of phages wV8 (top) and Felix O1 (bottom)**. The contiguous black boxes under the phage names represent the position of the genes. Regions of nucleotide similarity are indicated by the height of the coloured bars while those regions which are dissimilar are in white. The positions of the tail fibre genes are indicated by green rectangles.

Whole genome comparisons were made at the DNA level using Mauve [16] and Advanced Pipmaker [17] and at the protein level using CoreGenes [18]. The latter program revealed that Felix O1 and wV8 share 92% of their proteins in common. Mauve analysis (Figure 3) reveals considerable sequence similarity between Felix O1 and wV8 with a few noticeable differences which centre at 11.9, 26.7 52.3, and 60 kb on the Felix O1 genome. The presence of heterologous sequences within these phage genomes is completely in accord with the evolution of the viruses via horizontal gene transfer [19].

Based upon an extensive analysis of relationships between prokaryotic viruses (Lavigne R, Summer EJ, Seto D, Mahadevan P, Nilsson AS, Ackermann H-W *et al*.: Classification of *Myoviridae *bacteriophages using BLASTP-tools: submitted) this level of similarity indicates that wV8 should be classified into the newly proposed genus,"Felix O1 viruses", along with *Erwinia amylovora *phage φEa21-4.

## Conclusion

*E. coli *O157:H7-specific phage wV8 is a member of the *Myoviridae *and is closely related to the *Salmonella*-specific phage, Felix O1. Their tail fibre proteins show a unique pattern of sequence relationship.

## Competing interests

The authors declare that they have no competing interests.

## Authors' contributions

RA originally isolated phage V8. AMK assisted in the annotation and prepared the manuscript, EJL and SO propagated and purified the phage, and together with YMS contributed to the proteome analysis. HWA and DMM carried out the electron microscopy and AV annotated the genome. AM and EJL carried out the host range studies. TW and RPJ conceived and conducted the initial host range studies, other selection procedures and the animal studies for evaluation of wV8 as one of a cocktail of phages for control of *E. coli *O157:H7 in cattle. RPJ also contributed to manuscript preparation.

## Supplementary Material

Additional file 1 **ClustalW alignment of the tail fibre proteins of phages wV8 and Felix O1**. Alignments were carried out at EBI . Residues are indicated with a star (*) if identical, a colon (:) if conserved; and a period (.) if related. Click here for file
