# Supplementary material for: The genome and proteome of a virulent Escherichia coli O157:H7 bacteriophage closely resembling Salmonella phage Felix O1
Source: Virol J. 2009 Apr 20;6:41. doi: 10.1186/1743-422X-6-41 (PMC2674420; doi:10.1186/1743-422X-6-41)
Supplement: Additional file 1 — ClustalW alignment of the tail fibre proteins of phages wV8 and Felix O1. Alignments were carried out at EBI . Residues are indicated with a star (*) if identical, a colon (:) if conserved; and a period (.) if related. [file 1743-422X-6-41-S1.doc]

| **wV8 MADYKLSQLNSIDTIRSEDLLHIRVKKRPEMLGDEDRRMTYQDFLASFKLERFVQIAGST 60**  **Felix MADYKLSELNSIDTIRSDDLLHVRVKKRPEMLGDEDRRMTYQDFLASFKLERFVQIAGST 60**  *********:*********:****:***************************************  **wV8 MTGDLGIVKLLYGGKAVFDPTGSSEITIGDVLKTFKLNASGLKLTIADASRSATVYHTLN 120**  **Felix MTGDLGIVKLLYGGKAVFDPTGSSEITMGDVLKTFKINANGLKLTIADASRSATVYHTLN 120**  *****************************:********:**.**********************  **wV8 KPSPNELGMRTNEENDARYARLAITNTFSGTQNIQGDANLLRLRNQNANNAQYIEGVNLD 180**  **Felix KPSPNELGMRTNEENDARYARLAVTNTFSGTQNIQGDVNLLRLRNQNANNAQYIEGVDLD 180**  *************************:*************.*******************:****  **wV8 GSARWWVGIGSNGSDEVKLCNNKYNSVLTVASNISVNKSLAITGQVQPSDFSNLDARYFT 240**  **Felix GSARWLVGISKNGSDAVQLYNNKYDSALTIASNISVNKSLAITGQVQPSDFSNLDARYFT 240**  ******* ***..**** *:* ****:*.**:********************************  **wV8 QTAANQRFAQLAGNNTFTGANTFT-NLVAKKNANAITLQNTDANTALYILGKKSDGTNKW 299**  **Felix QTVANQRFAQLAANNNFTGTNTFSRNLTIISDSAALRLKNATS-SSLFVQGVDSQNTNRW 299**  ****.*********.**.***:***: **. .:: *: *:*: : ::*:: * .*:.**:***  **wV8 YVGTDSDETRLNIYNYLTGSQVSLG-TTIGINKTVQITGQVQPSDWANIDSRYIPVATLS 358**  **Felix YVGNGDNTASVLLHNYVHGSNIRLDNGYISVNQNFRITGQVQPSDFSNIDSRYIPAATLS 359**  *****...: : : ::**: **:: *. *.:*:..:*********::********.******  **wV8 TIARTNARNTFNGVQTVVTDNEGLIVKNSTQNRPLYIRGVDTTNVSRWWIGVGGADTNDV 418**  **Felix TIARTNAQNTFNGAQTVVSDGEGLVIKNSTQNRPLYIRGKDAANVSRWWLGVGDPNSTDV 419**  *********:*****.****:*.***::************* *::******:***..::.****  **wV8 TLNNSYSGTQLVLGNTTSYINKTLTIAGQVQPSDFSNLDARYFTQSASDSRYLRIRS--T 476**  **Felix ALNNSFSGTQLILGNSSASINKTLTLAGQIQPSDFSNLDARYYTQSTANSRYMLAYSSGT 479**  **:****:*****:***::: ******:***:************:***:::***: * ***  **wV8 SFNVGNTDKWAKIATVVMPQSASTAVIEVFGGSGFNINTPNQAGKCEIVLRTSNNNPKGL 536**  **Felix GTEVGDSD--------GIAWNAKTGLYNVTGYSG---------GSTQLVFQMYQGAS--- 519**  **. :**::* :. .*.*.: :* * ** *. ::*:: :. .**  **wV8 NVVAWRTSENTIVRDIGYVNTSGDTYDIYYLAGTYQNSTTTRVQSSSNASVQLFEVPQTF 596**  **Felix -------------------STPSAQLKFNYRNGGFWYRS----------SRDGFGFEEDF 550**  **.*.. .: * * : : * : * . : ***  **wV8 DDAPQGIVKGTIAKYYTSLQKPTPSDIGAYTKAETDQKIAEAISDSTDLNKIYPVGIVTW 656**  **Felix T------------QIYTEKYKPTPSAIGAYTKAETDQKIAEAISDSTDLNKIYPVGIVTW 598**  **: **. ***** ************************************  **wV8 FNSNVNPNTALPGLTWTYLNNGVGRTIRIAAANGSDVATTGGSDSVTLSVGNLPSHTHSF 716**  **Felix FNSNVNPNTALPGLTWTYLNNGVGRTIRIAAANGSDVATTGGSDSVTLSVGNLPSHTHSF 658**  ****************************************************************  **wV8 SATTSSFDYGTKTSSTTGNHNHNRGTMEITGSFGYFRSDASSFYTASGAFYLGSQAGSKG 776**  **Felix SATTSSFDYGTKTTNTTGAHTHS-----VSGST----NNTGAHTHTFGGRYGGDSIGGKH 709**  ***************:.*** *.*. ::** .::.:. : *. * *.. *.***  **wV8 YTGNNFTNGIP-VNFNASRNWSGVTNTTGNHSHTVGIGAHSHTVSGNTGGTGSGSAFSVT 835**  **Felix RVHVSGTEQVSSVAGDHSHTVYGTAASNGNHAHTVGIGAHSHTVSGNTGGTGSGSAFSVT 769**  **. . *: :. * : *:. *.: :.***:******************************  **wV8 NQFYKLMAWVRTA 848**  **Felix NQFYKLMAWVRTA 782**  ***************** |
| --- |
